# The Complete Chloroplast Genome Sequence of *Callisia fragrans* (Lindl.) Woodson (Commelinaceae)

**DOI:** 10.1002/ece3.71402

**Published:** 2025-05-14

**Authors:** Khang Vo‐Tan, Van Truong Thi Bich, Men Tran Thanh, Tai Tran Tien, Hoang Dang Khoa Do, Ngoc‐Van Thi Nguyen

**Affiliations:** ^1^ Can Tho University Can Tho Vietnam; ^2^ Pham Ngoc Thach University of Medicine Ho Chi Minh Vietnam; ^3^ NTT Hi‐Tech Institute Nguyen Tat Thanh University Ho Chi Minh Vietnam; ^4^ Can Tho University of Medicine and Pharmacy Can Tho Vietnam

**Keywords:** Commelinoideae, comparative genomics, invasive species, spiderworts

## Abstract

*Callisia fragrans* (Lindl.) Woodson (Commelinaceae) is an invasive species in Vietnam but exhibits ornamental and potential medicinal values. However, the genomic data of *C. fragrans* have not been discovered. In this study, we employed the Illumina sequencing platform to complete the chloroplast genome of *C. fragrans* , which was 163,887 bp in length. This quadripartite genome consisted of a large single copy region of 90,751 bp, a small single copy region of 18,684 bp, and two inverted repeat regions of 27,226 bp each. Additionally, there were 79 protein‐coding genes, 30 transfer RNA genes, and four ribosomal RNA genes in the chloroplast genome of *C. fragrans* . Comparative genomic analysis revealed a conserved pattern of genome structure and gene content among *Callisia* species. However, in contrast to the pseudogenization of *accD* and *rpoA* in *C. repens* and *C. insignis*, these genes were intact in *C. fragrans* . Comparative genomic analysis revealed seven variable regions in the chloroplast genomes of three *Callisia* species, including *rps16‐trnQ_UUG*, *psbI‐trnG_UCC*, *rpoB‐psbM*, *trnP_UGG‐rpl33*, *ndhF‐trnL_UAG*, *rps15‐ycf1*, and *ycf1*. Phylogenetic analysis indicated the monophyly of *Callisia* species and a close relationship between *C. fragrans* and *C. repens* . This study provides initial data of the chloroplast genome for further genomic studies examining genetic populations, phylogeny, and molecular markers of *C. fragrans* and related species in Commelinaceae.

## Graphical abstract

This research reported the complete chloroplast genome of *Callisia fragrans* , an ornamental and medicinal plant in the Commelinaceae family. The comparative genomic analysis revealed a unique feature of the *C. fragrans* chloroplast genome within the *Callisia* genus.

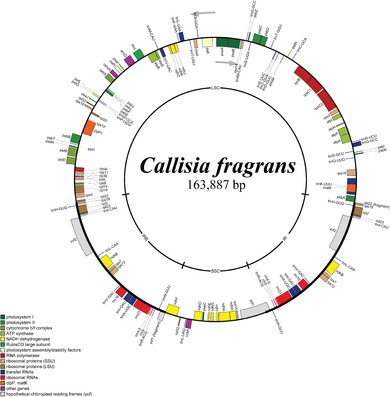

## 1 Introduction

*Callisia fragrans* (Lindl.) Woodson is a monocotyledonous, perennial herbaceous plant which belongs to the *Callisia* Loefl. genus, Commelinaceae family. This plant is native to Mexico and intentionally introduced to other continents for ornamental or medicinal purposes (Graveson 2012). Ethnobotanical surveys have reported that *C. fragrans* preparations can be used to treat joint problems, reduce body pain and swelling, or as a natural detox supplement (Sõukand et al. 2017; Pranskuniene et al. 2019; Sengthong et al. 2024). The chemical composition of *C. fragrans* includes carotenoids, xanthophyll, carbohydrates, anthraquinone, scopoletin, coumarin, flavonoids, acid phenolic, and phyto‐steroids (Chernenko et al. 2007; Olennikov et al. 2008, 2010; Hang et al. 2015). Studies have shown that *C. fragrans* has various pharmacological traits, such as antioxidant, anti‐inflammation, anti‐bacterial, anti‐herpetic, anti‐hypertensive, and anti‐tumor (Tan et al. 2014; El Sohafy et al. 2021). *C. fragrans* was introduced to Vietnam due to a belief that this plant is a hidden cure for cancers. Consequently, *C. fragrans* has been widely cultivated in Vietnam. As *C. fragrans* is a non‐native species in Vietnamese flora, botanical analysis or pharmaceutical analysis to identify this species can be problematic due to lacking observations and experiences. There is an urgent need for a molecular biological approach to create a standardized medicinal crop, as well as facilitating a control strategy of this invasive species in Vietnam.

The chloroplasts are plant‐specific organelles which conduct photosynthesis to provide energy for plants and algae (Gray 1989). As they have their own genetic replication mechanism, transcribe their own genome, and carry out maternal inheritance, these plastid DNA are highly conserved in gene order and gene content. The chloroplast genome size often ranges from 100 to 160 kb, which can serve as a valuable source of reference for molecular phylogeny and molecular ecology research (Olmstead and Palmer 1994). A good understanding of the chloroplast genome can provide necessary DNA sequences to authenticate a plant at the species level.

In this study, we assembled and analyzed the complete chloroplast genome of *C. fragrans* for the first time by using the Illumina high‐throughput sequencing technology. The features of the *C. fragrans* chloroplast genome were also compared with those of other *Callisia* species to explore the variations. Additionally, the phylogenetic relationship inferred from 77 protein‐coding genes of the chloroplast genome was reconstructed to explore the relationships between *C. fragrans* and related species in Commelinaceae. The outcomes of this study added new genomic data of the *Callisia* genus and provided necessary molecular data for further studies examining molecular evolution among Commelinaceae members.

## 2 Materials and Methods

**FIGURE 1:**
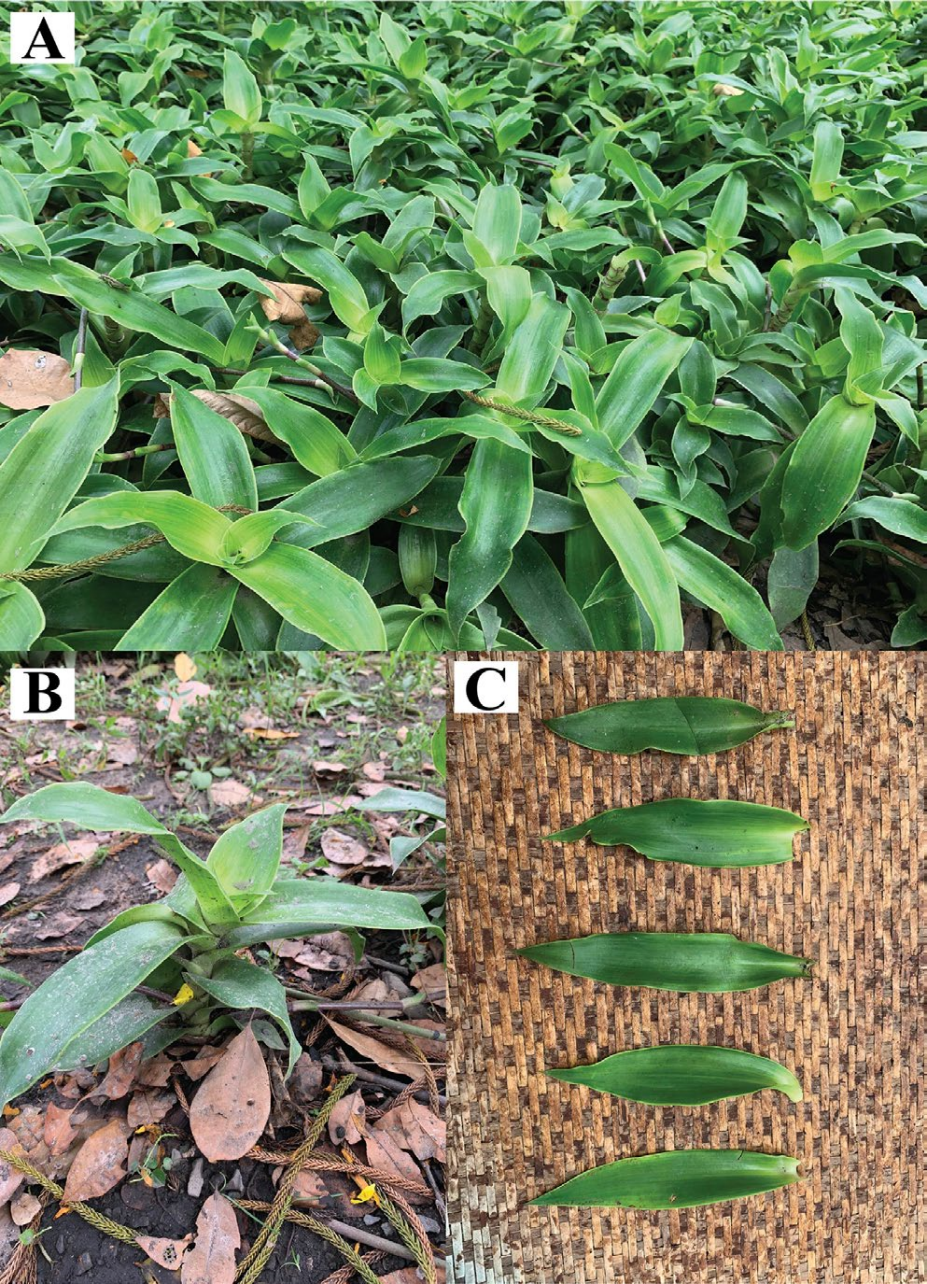
Illustration of *Callisia fragrans* in the field. (A) The population in the field. (B) The whole plant of an individual. (C) The leaves. Leaves are oblong to lanceolate‐oblong and arranged spirally. Stems are fleshy and up to one meter tall and have stolons at base. Photo taken by Khang Vo‐Tan in Hoc Mon district, Ho Chi Minh City, Vietnam.

The healthy leaves of *C. fragrans* were collected at Hoc Mon District, Ho Chi Minh City, Vietnam (geographical location 10°54′31.6'N 106°35′52.7'E, Figure 1). The collected leaves were then stored in a deep freezer at −81°C for further experiments. The specimen of *C. fragrans* was deposited in Pham Ngoc Thach University of Medicine under voucher number UPNT‐VHA‐202501001 (contact person: Mr. Khang Vo‐Tan, email: votankhang@pnt.edu.vn). The collected leaves were used to extract total genomic DNA using a DNeasy Plant Pro Kit (Qiagen, USA). The high‐quality DNA sample, which exhibited a minimum concentration of 100 ng/μL and a clear band on the gel without smear using NanoDrop One Microvolume UV–Vis Spectrophotometer (Thermo Fisher Scientific, USA) and 1% agarose gel electrophoresis, respectively, was used for sequencing process with the Nextseq500 (Illumina, USA) to generate a dataset of 150 bp paired‐end reads.

Among the raw reads, the reads with a phred score ≤ 20, reads containing N bases, and reads shorter than 100 bp were eliminated using fastp v0.23.4 with default settings (Chen et al. 2018). The remaining reads were used to assemble the complete chloroplast genome using NOVOPlasty v4.3.5 with the reference genome of *Callisia repens* (accession number MW617982) and default settings (Dierckxsens et al. 2017). The Geseq with default settings was used to annotate the newly complete chloroplast genome of *C. fragrans* (Tillich et al. 2017). The Geneious Prime v2024.0.1 and tRNAscan‐SE were used to verify protein‐coding regions for start and stop codons and tRNA composition, respectively (www.geneious.com; [Chan and Lowe 2019]). The OGDRAW was employed to create the map of *C. fragrans* plastome with default settings (Greiner et al. 2019).

For comparative analyses, the complete chloroplast genomes of *C. fragrans* , *C. insignis* , and *C. repens* were used. The boundaries among LSC, SSC, and IR regions were characterized using Geneious Prime v2024.0.1, of which the function of Find repeat for a minimum length of 2000 bp was used to locate two IR regions. For nucleotide diversity analysis, whole chloroplast genomes of three *Callisia* species were aligned using MUSCLE v5 before calculating Pi values with a window length of 1000 bp and a step size of 200 bp using DnaSP v6 (Edgar 2004; Rozas et al. 2017). To calculate nonsynonymous (Ka) and synonymous (Ks) substitution rates, 79 protein‐coding regions of three *Callisia* species were aligned to those of *Gibasis geniculata* (MW617987) using MAFFT v7 (Katoh 2002). Then, the Ka, Ks, and Ka/Ks ratios were estimated using DnaSP v6. Additionally, single nucleotide polymorphism (SNP) and insertion/deletion events (indels) in chloroplast genomes between *C. fragrans* and its closest counterpart inferred from phylogenetic analysis were identified using Geneious Prime v2024.0.1 with default settings of Find variations/SNPs option.

For phylogenetic analysis, complete chloroplast genomes of 27 species were downloaded from the NCBI database (https://www.ncbi.nlm.nih.gov/), which includes 26 species of Commelinaceae and *Zingiber officinale* (accession number MH161428) of Zingiberaceae as outgroups (Table 1). There were two datasets for elucidating phylogenetic relationships between *C. fragrans* and related species. For the first dataset, a total of 77 protein‐coding genes (except *accD* and *rpoA* due to pseudogenization) of 28 species were extracted from the chloroplast genomes and aligned using MAFFT with default settings (Katoh 2002). The second dataset included whole chloroplast genome sequences which were aligned using MUSCLE and trimmed using trimAl v1.5.0 with default settings (Edgar 2004; Capella‐Gutiérrez et al. 2009). The jModeltest2 was used to find the best substitution model with default settings for the aligned sequences (Darriba et al. 2012). As a result, the GTR + I + G was selected as the best substitution model for the two datasets of 28 examined species. For conducting phylogenetic analysis, the maximum likelihood (ML) and Bayesian inference (BI) methods were used. For the ML method, IQ‐TREE2 was used with the GTR + I + G substitution model, 1000 replications for bootstrap analysis, and other default settings (Minh et al. 2020). For BI methods, MrBayes v3.2.7a was used with the GTR + I + G model, 1,000,000 generations, and discard of 25% of samples (Ronquist et al. 2012). The Figtree v1.4.4 was used to illustrate the phylogenetic trees (http://tree.bio.ed.ac.uk/software/figtree/).

**TABLE 1:** List of species for phylogenetic analysis.

Family	Subfamily	Species	Accession number
Commelinaceae	Commelioideae	*Murdannia edulis*	MW617988
		*Cyanotis ciliata*	MK133255
		*Cyanotis speciosa*	OP758350
		*Amischotolype hispida*	MW617981
		*Weldenia candida*	MW617995
		*Geogenanthus poeppigii*	MW617986
		*Siderasis fuscata*	MW617992
		*Dichorisandra thyrsiflora*	MW617985
		*Palisota barteri*	MW617989
		*Tradescantia pallida*	MT527961
		*Streptolirion volubile*	MW617993
		*Cochliostema odoratissimum*	MW617983
		*Spatholirion longifolium*	OP758352
		*Pollia japonica*	MW145132
		*Tradescantia virginiana*	MW617994
		*Tradescantia ohiensis*	MZ203134
		*Callisia insignis*	OP758347
		*Aneilema beniniense*	OP758345
		*Callisia repens*	MW617982
		*Callisia fragans*	PQ834833
		*Gibasis geniculata*	MW617987
		*Rhopalephora scaberrima*	MW617991
		*Commelina caroliniana*	OR936140
		*Commelina benghalensis*	OQ354383
		*Commelina communis*	MW617984
		*Tinantia pringlei*	OP758353
	Cartonematoideae	*Cartonema parviflorum*	OP758348
Zingiberaceae		*Zingiber officinale*	MH161428

## 3 Results and Discussion

**FIGURE 2:**
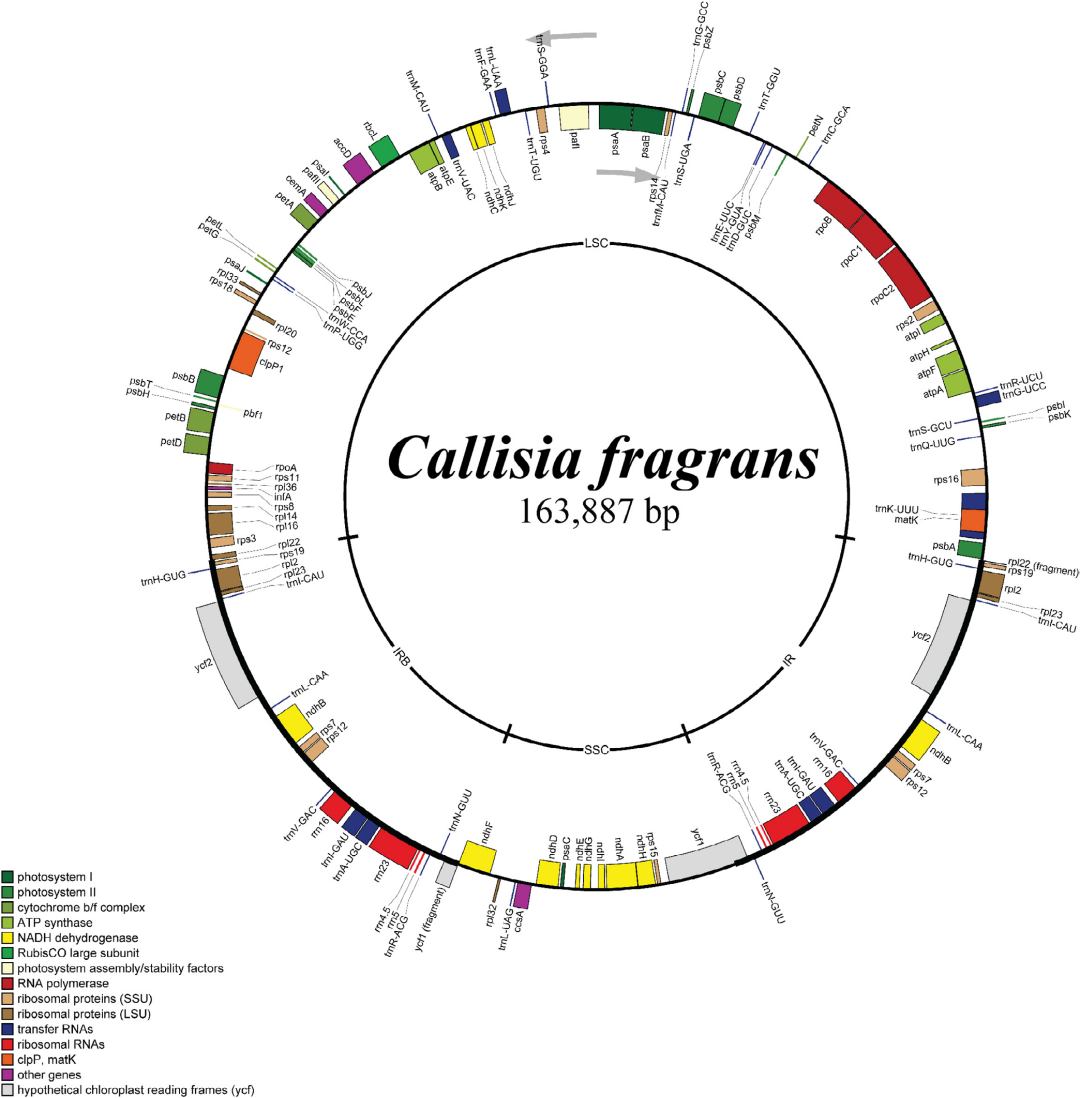
Map of the chloroplast genome of *Callisia fragrans* . The arrows indicated the translation directions of inner and outer genes. The inner circle indicates four regions of the chloroplast genome. IRA and IRB: inverted repeat regions; LSC: large single copy; SSC: small single copy.

**FIGURE 3:**
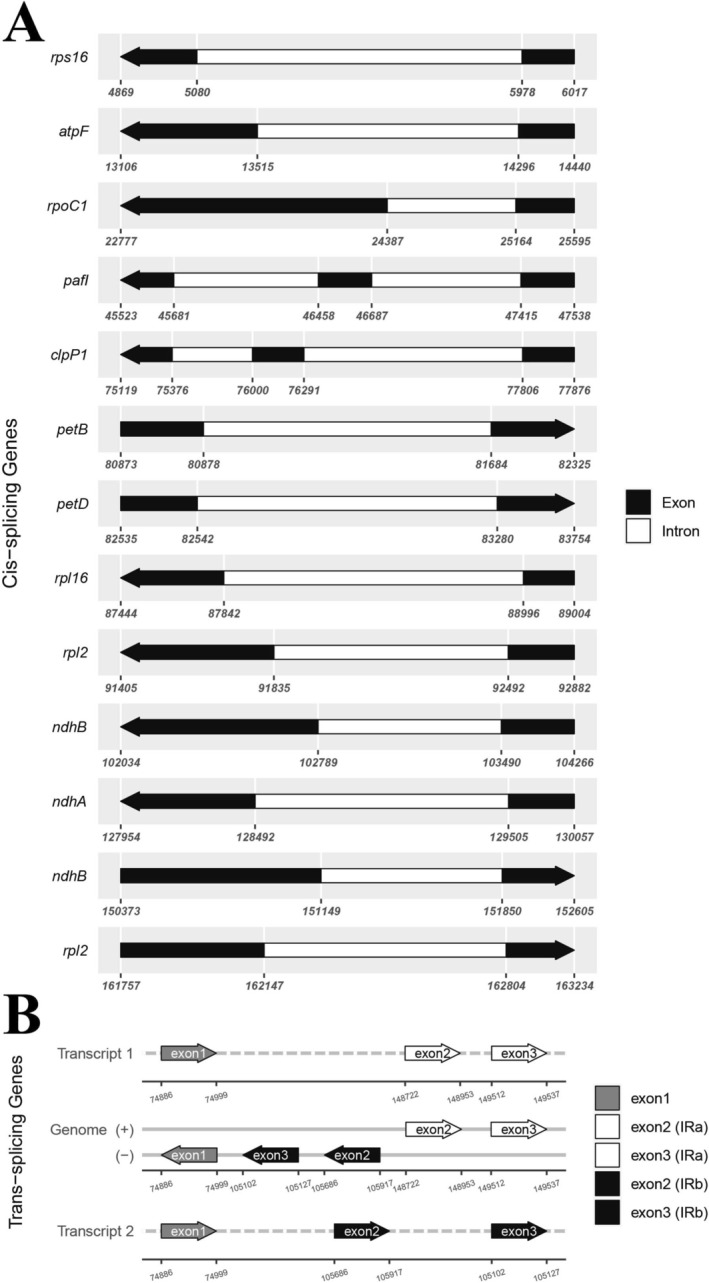
Schematic map of the *cis*‐ and *trans*‐splicing genes in the chloroplast genomes of *Callisia fragrans* . (A) The *cis*‐splicing genes. (B) The *trans*‐splicing genes.

The assembly process resulted in a circular genome of 163,887 bp, of which the average coverage was 500x (Figures 2 and S1). The complete chloroplast genome of *C. fragrans* consisted of a large single copy (LSC) of 90,751 bp, a small single copy of 18,684 bp, and two inverted repeat (IR) regions of 27,226 bp each. The overall guanine and cytosine (GC) content was 35.8%, whereas those for LSC, SSC, and IR regions were 33%, 30%, and 42.4%, respectively. Furthermore, the plastome of *C. fragrans* contained 79 protein‐coding genes, 30 transfer RNA genes, and four ribosomal RNA genes (Table 2). Among the annotated genes, there were two genes (*pafI* and *clpP1*) containing two introns and nine genes having one intron (including *rps16*, *atpF*, *rpoC1*, *petB*, *petD*, *rpl16*, *rpl2*, *ndhB*, and *ndhA*; Table 2, Figure 3A). The *rps12* gene was trans‐splicing with exon1 in the LSC region and exon2 and exon3 in the IR regions (Figure 3B). Because of the presence of IR regions, there were 21 duplicated genes in the IR regions, including *rrn4.5*, *rrn5*, *rrn16*, *rrn23*, *trnA‐UGC*, *trnH‐GUG*, *trnL‐CAA*, *trnI‐GAU*, *trnI‐CAU*, *trnN‐GUU*, *trnR‐ACG*, *trnV‐GAC*, *rpl2*, *rpl22*, *rpl23*, *rps7*, *rps12*, *rps19*, *ndhB*, *ycf1*, and *ycf2*, of which *ycf1* and *rpl22* were incompletely duplicated.

**TABLE 2:** Gene composition of *Callisia fragrans* chloroplast genome.

Groups of genes	Name of genes
Ribosomal RNAs	*rrn4.5* ^a^, *rrn5* ^a^, *rrn16* ^a^, *rrn23* ^a^
Transfer RNAs	*trnA‐UGC* ^a^, ^b^, *trnC‐GCA*, *trnD‐GUC*, *trnE‐UUC*, *trnF‐GAA*, *trnG‐UCC* ^b^, *trnG‐GCC*, *trnH‐GUG*, *trnI‐GAU* ^a^, ^b^, *trnK‐UUU* ^b^, *trnL‐CAA* ^a^, *trnL‐UAA* ^b^, *trnL‐UAG*, *trnfM‐CAU*, *trnI‐CAU* ^a^, *trnM‐CAU*, *trnN‐GUU* ^a^, *trnP‐UGG*, *trnQ‐UUG*, *trnR‐ACG* ^a^, *trnR‐UCU*, *trnS‐GCU*, *trnS‐GGA*, *trnS‐UGA*, *trnT‐GGU*, *trnT‐UGU*, *trnV‐GAC* ^a^, *trnV‐UAC* ^b^, *trnW‐CCA*, *trnY‐GUA*
Large units of ribosome	*rpl2* ^a^, ^b^, *rpl14*, *rpl16* ^b^, *rpl20*, *rpl22* ^a^, *rpl23*, *rpl32*, *rpl33*, *rpl36*
Small units of ribosome	*rps2*, *rps3*, *rps4*, *rps7* ^a^, *rps8*, *rps11*, *rps12* ^a^, ^c^, *rps14*, *rps15*, *rps16*, *rps18*, *rps19* ^a^
RNA polymerase	*rpoA*, *rpoB*, *rpoC1* ^b^, *rpoC2*
Translational initiation factor	*infA*
Subunit of photosystem I	*psaA*, *psaB*, *psaC*, *psaI*, *psaJ*, *pafI* ^c^, *pafII*
Subunit of photosystem II	*psbA*, *psbB*, *psbC*, *psbD*, *psbE*, *psbF*, *psbH*, *psbI*, *psbJ*, *psbK*, *psbL*, *pbfI*, *psbM*, *psbT*, *psbZ*
Subunit of cytochrome	*petA*, *petB* ^b^, *petD* ^b^, *petG*, *petL*, *petN*
Subunit of ATP synthases	*atpA*, *atpB*, *atpE*, *atpF* ^b^, *atpH*, *atpI*
Large unit of Rubisco	*rbcL*
Subunit of NADH dehydrogenase	*ndhA* ^b^, *ndhB* ^a^, ^b^, *ndhC*, *ndhD*, *ndhE*, *ndhF*, *ndhG*, *ndhH*, *ndhI*, *ndhJ*, *ndhK*
Maturase	*matK*
Envelope membrane protein	*cemA*
Subunit of acetyl‐CoA	*accD*
C‐type cytochrome synthesis gene	*ccsA*
ATP‐dependent protease subunit P	*clpP1* ^c^
Hypothetical proteins and conserved reading frames	*ycf* ^a^, *ycf2* ^a^

^a^ Duplicated gene in the IR region.

^b^ Genes containing a single intron.

^c^ Genes containing two introns.

**FIGURE 4:**
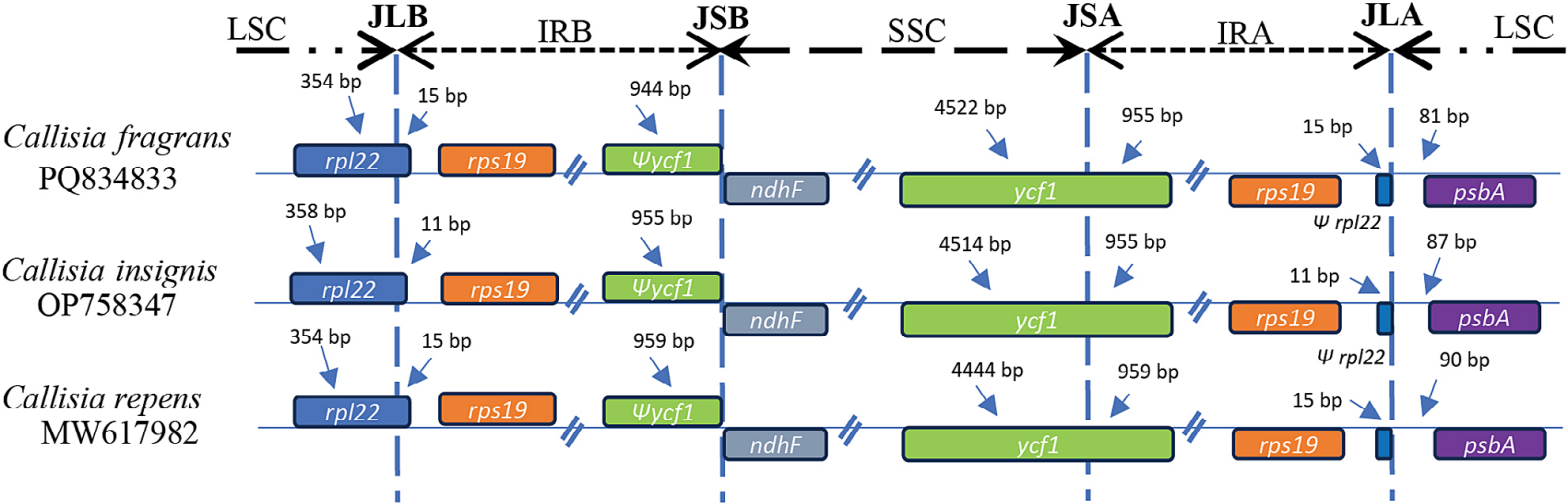
The junctions among LSC, SSC, and IR regions of three *Callisia* choroplast genomes. IRA and IRB: inverted repeat regions; JLA: junction between LSC and IRA regions; JLB: junction between LSC and IRB regions; JSA: junction between SSC and IRA regions; JSB: junction between SSC and IRB regions; LSC: large single copy; SSC: small single copy.

Further comparative analysis revealed a slight change in the length of the chloroplast genome among three *Callisia* species, of which the total length ranged from 161,854 bp to 163,887 bp (Table 3). Consequently, the lengths of LSC, SSC, and IR regions fluctuated from 89,446 bp to 90,751 bp, from 18,252 bp to 18,786 bp, and from 27,078 bp to 27,226 bp, respectively. A similar trend was also observed in the GC content of the three examined chloroplast genomes. However, the number of protein‐coding genes, tRNA genes, and rRNA genes was the same among the three *Callisia* species. The boundaries among LSC, SSC, and IR regions were in the *rpl22* gene for the LSC/IR region and the *ycf1* gene for the SSC/IR junction (Table 3, Figure 4). However, the locations of junction sites were different in the three *Callisia* species. For example, the SSC/IR junction sites were 955 bp, 959 bp, and 944 bp from the start codon of *ycf1* in *C. insignis* , *C. repens* , and *C. fragrans* , respectively. Similarly, the LSC/IR junction site was 11 bp from the start codon of *rpl22* in *C. insignis* and 15 bp from the start codon of *rpl22* in *C. repens* and *C. fragrans* (Figure 4). The features of the *C. fragrans* chloroplast genome were similar to previously published chloroplast genomes of Commelinaceae regarding genome structure and gene content (Jung et al. 2021). However, in contrast to the pseudogenization of *accD* and *rpoA* genes in the Commelinoideae subfamily (including *C. repens* and *C. insigns*; [Jung et al. 2021]), these two genes were intact in *C. fragrans* . This difference indicated a unique evolutionary path of *C. fragrans* , which needs further confirmation with other species in the *Callisia* genus and Commelinaceae family.

**TABLE 3:** Features of chloroplast genomes among *Callisia* species.

Species	*Callisia insignis*	*Callisia repens*	*Callisia fragrans*
Accession number	OP758347	MW617982	PQ834833
Total length (bp)	162,850	161,854	163,887
Total % GC	35.9	36	35.8
LSC length (bp)	89,860	89,446	90,751
LSC % GC	33.2	33.2	33
SSC length (bp)	18,786	18,252	18,684
SSC % GC	30.1	30.3	30
IR length (bp)	27,102	27,078	27,226
IR % GC	42.3	42.5	42.4
Protein‐coding gene	79	79	79
tRNAs	30	30	30
rRNAs	4	4	4
LSC/IR junction	*rpl22* (11 bp)	*rpl22* (15 bp)	*rpl22* (15 bp)
SSC/IR junction	*ycf1* (955 bp)	*ycf1* (959 bp)	*ycf1* (944 bp)

**FIGURE 5:**
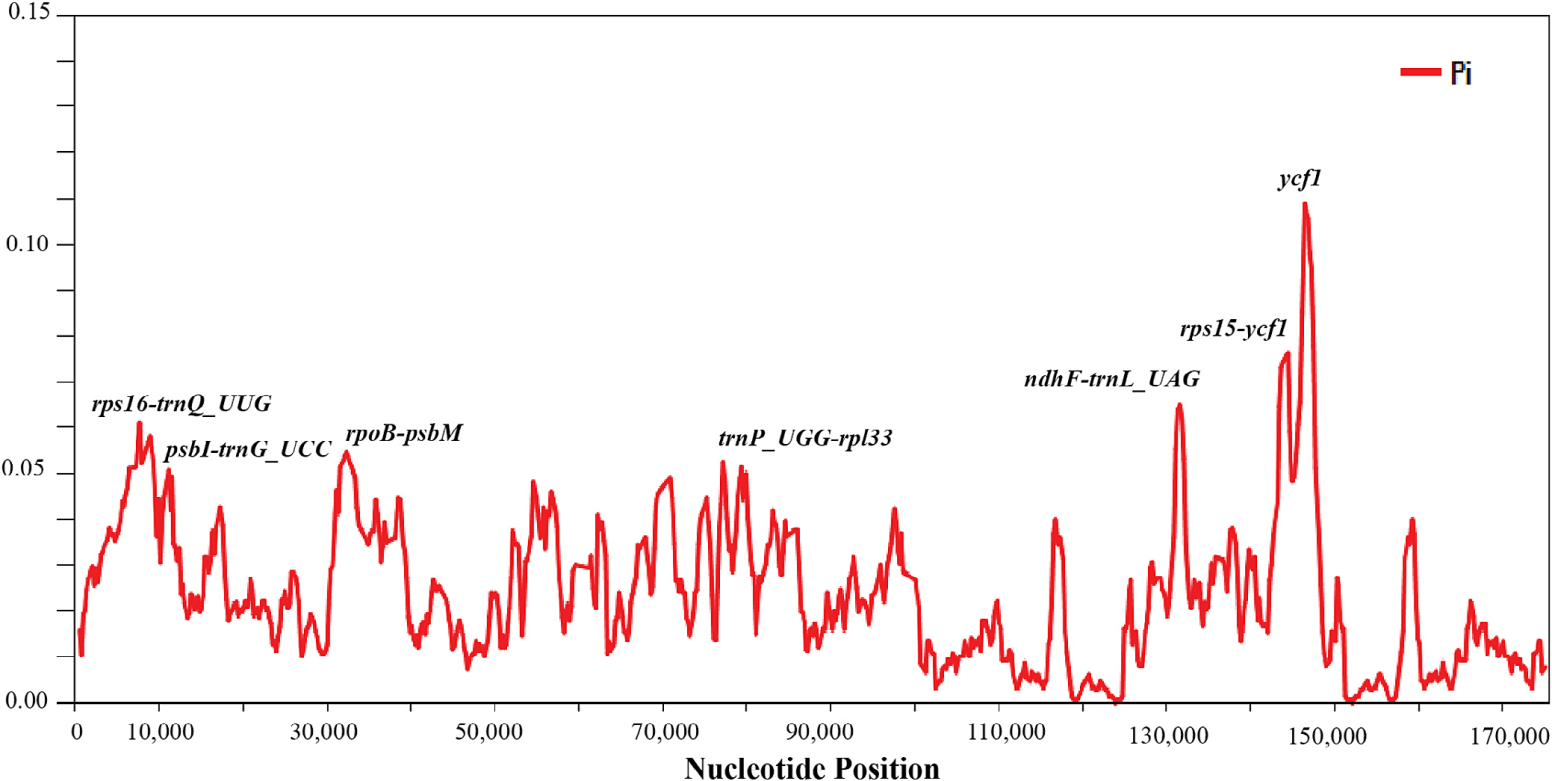
Nucleotide diversity among three *Callisia* chloroplast genomes.

**FIGURE 6:**
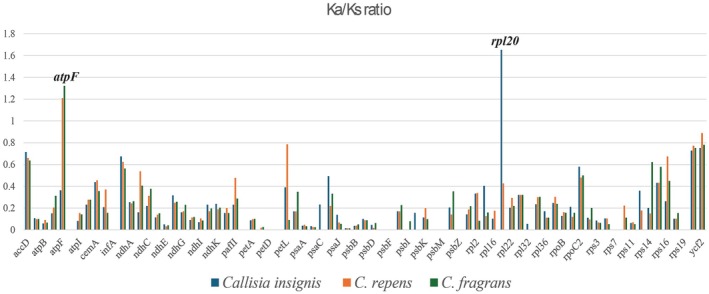
Non‐synonymous (*K*a)/synonymous (*K*s) ratios among 79 protein‐coding regions of three *Callisia* chloroplast genomes.

The nucleotide diversity revealed seven highly variable regions among *Callisia* cpDNAs, including *rps16‐trnQ_UUG*, *psbI‐trnG_UCC*, *rpoB‐psbM*, *trnP_UGG‐rpl33*, *ndhF‐trnL_UAG*, *rps15‐ycf1*, and *ycf1* with Pi values > 0.05 (Figure 5). Previously, nucleotide diversity among coding regions of 16 Commelinoideae chloroplast genomes revealed nine genes with Pi values > 0.05 such as *ndhF*, *matK*, *rps3*, and *ycf1* (Jung et al. 2021). Although the previous study did not include noncoding regions for nucleotide diversity analysis, its outcome indicated that *ycf1* of Commelinoideae had high nucleotide diversity (Pi values = 0.09543). Similarly, the same pattern of *ycf1* was found in three *Callisia* species with Pi value = 0.109, suggesting *ycf1* as a potential candidate for mining molecular markers in *Callisia* and related taxa in Commelinaceae. Further analysis of Ka/Ks ratios indicated that most genes in the chloroplast genome of *Callisia* species exhibited negative selection (Ka/Ks ratio < 1) (Figure 6). However, the *atpF* gene of *C. repens* and *C. fragrans* and the *rpl20* gene of *C. insignis* displayed positive selection (Ka/Ks ratio > 1). Previously, the Ka/Ks ratio of protein‐coding genes had not been calculated in Commelinaceae, but it has been widely observed in other land plants such as Theaceae and Actinidaceae (Gladysheva‐Azgari et al. 2023; Fan et al. 2025). Particularly, in Theaceae, only four genes including *matK*, *ndhB*, *rpoC1*, and *ycf1* showed a positive selection (Fan et al. 2025). Similarly, only the *ycf2* gene in Actinidaceae was under positive selection (Gladysheva‐Azgari et al. 2023). In the current study, the majority of genes were under negative selection, suggesting the stability of genes in chloroplast genomes of *Callisia* (Figure 6). However, genes that are under positive selection are different in the chloroplast genomes of each family and need further investigation.

**FIGURE 7:**
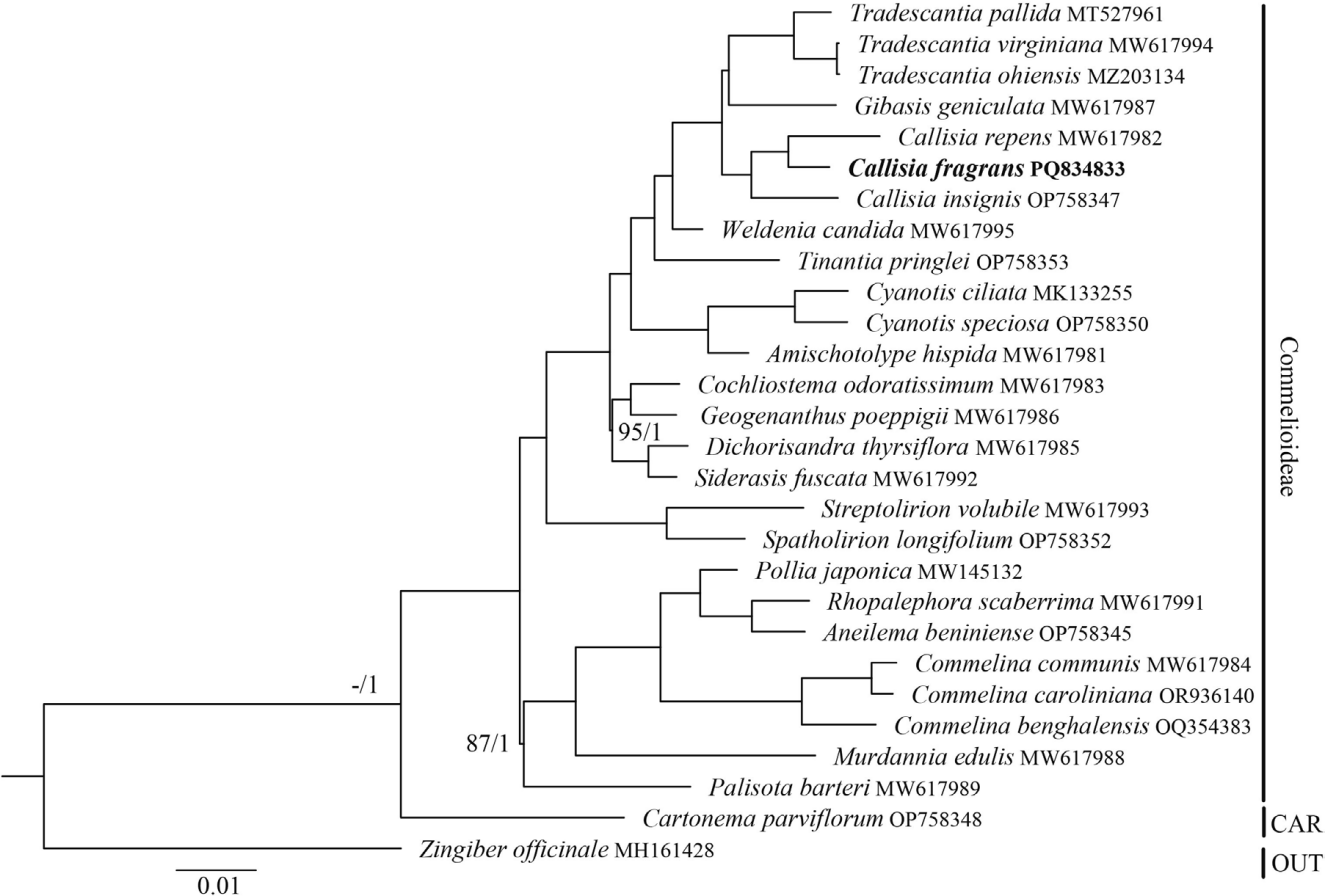
Phylogenetic relationship of *Callisia fragrans* and related species inferred from 77 protein‐coding genes using maximum likelihood and Bayesian inference methods. Only bootstrap values smaller than 100 and posterior probability under 1 were shown at the nodes. The bold italic name means the newly generated chloroplast genome in this study. CAR: Cartonematoideae; OUT: outgroup.

For phylogenetic analysis, the ML and BI methods resulted in the same topology of phylogenetic trees inferred from two datasets (Figures 7 and S2, Table S1). The monophyly of Commelinoideae was revealed with high support values (bootstrap value = 100, posterior probability = 1). Within Commelinoideae, *Callisia* species formed a clade with *Tradescantia* and *Gibasis* species. Previously, a similar relationship among *Callisia*, *Tradescantia*, and *Gibasis* was also reported in different phylogenetic studies based on nuclear and chloroplast DNA sequences (Burns et al. 2011; Zuntini et al. 2021; Lee et al. 2022). Among the three examined *Callisia* species, *C. fragrans* was close to *C. repens* . Further comparative genomic analysis between *C. fragrans* and *C. repens* revealed 533 indels, ranging from 1 bp to 1045 bp, of which 33 indels were located in coding regions of *accD*, *ccsA*, *rpoC2*, *ycf1*, and *ycf2* (Table S2). Additionally, 2603 SNPs were found in cp genomes of *C. fragrans* and *C. repens* , of which 1006 and 1597 SNPs were found in coding regions and noncoding regions, respectively (Table S3). These results demonstrated variations of cp genomes between *C. fragrans* and *C. repens* , albeit these two species exhibited a close relationship. Although different studies examining morphology, cytogeography, and molecular data have been conducted, a few samples of *Callisia* were used (Hertweck and Pires 2014; Molgo et al. 2017; Pellegrini 2017). Therefore, further studies covering more samples of *Callisia* should be conducted to elucidate the evolutionary patterns.

## 4 Conclusion

In the current study, we reported the complete chloroplast genome of *C. fragrans* , of which the raw data were generated by the Illumina sequencing platform. The plastome of *C. fragrans* had a quadripartite structure and contained 79 protein‐coding genes, 30 tRNA genes, and four rRNA genes, which are similar to the previously published chloroplast genome of Commelinaceae. Phylogenetic analysis revealed the relationships among *Callisia*, *Tradescantia*, and *Gibasis* species within the Commelinoideae subfamily. The results of this study enlarge the genomic data of the *Callisia* genus and provide fundamental information for further genomic study of *Callisia* species and related taxa in Commelinaceae.

## Author Contributions

**Khang Vo‐Tan:** conceptualization (equal), data curation (lead), formal analysis (equal), writing – original draft (lead). **Van Truong Thi Bich:** data curation (supporting), methodology (supporting), software (supporting), visualization (equal), writing – original draft (supporting). **Men Tran Thanh:** conceptualization (equal), formal analysis (equal), methodology (supporting), software (lead), writing – review and editing (supporting). **Tai Tran Tien:** conceptualization (equal), formal analysis (equal), methodology (supporting), software (supporting), writing – review and editing (supporting). **Hoang Dang Khoa Do:** data curation (supporting), methodology (lead), writing – review and editing (supporting). **Ngoc‐Van Thi Nguyen:** conceptualization (equal), investigation (lead), writing – original draft (supporting), writing – review and editing (supporting).

## Conflicts of Interest

The authors declare no conflicts of interest.

## Supporting information


Figure S1.

Table S1.

Table S2.

Table S3.

## Data Availability

The complete chloroplast genome of *Callisia fragrans* was submitted to GenBank under accession number PQ834833. The raw sequencing data were also deposited to NCBI under the accession number PRJNA1209239.
