# Supplementary material for: The Complete Chloroplast Genome Sequence of Callisia fragrans (Lindl.) Woodson (Commelinaceae)
Source: Ecol Evol. 2025 May 14;15(5):e71402. doi: 10.1002/ece3.71402 (PMC12076062; doi:10.1002/ece3.71402)
Supplement: Supplementary file 1 — Figure S1. [file ECE3-15-e71402-s001.docx]

**The complete chloroplast genome sequence of *Callisia fragrans* (Lindl.) Woodson (Commelinaceae)**

Khang Vo-Tan^1,2^, Van Truong Thi Bich^1^, Men Tran Thanh^1^, Tai Tran Tien^2^, Hoang Dang Khoa Do ^3^, Ngoc-Van Thi Nguyen ^4.^*

^1^ Can Tho University, Can Tho City, Vietnam

^2^ Pham Ngoc Thach University of Medicine, Ho Chi Minh City, Vietnam

^3^ NTT Hi-Tech Institute, Nguyen Tat Thanh University, Ho Chi Minh City, Vietnam

^4^ Can Tho University of Medicine and Pharmacy, Can Tho City, Vietnam

*Corresponding author

Ngoc-Van Thi Nguyen

Can Tho University of Medicine and Pharmacy, Can Tho City, Vietnam

Email: ntnvan@ctump.edu.vn

**Supplementary Figure Legends**

**Supplementary Figure S1.** Coverage map of chloroplast genome of *Callisia fragrans*.

**Supplementary Figure S2.** Phylogenetic relationship of *Callisia fragrans* and related species inferred from whole chloroplast genome sequences using maximum likelihood and Bayesian Inference methods. Only bootstrap values smaller than 100 and posterior probability under 1 were shown at the nodes. The bold italic name means the newly generated chloroplast genome in this study. CAR: Cartonematoideae; OUT: Outgroup.


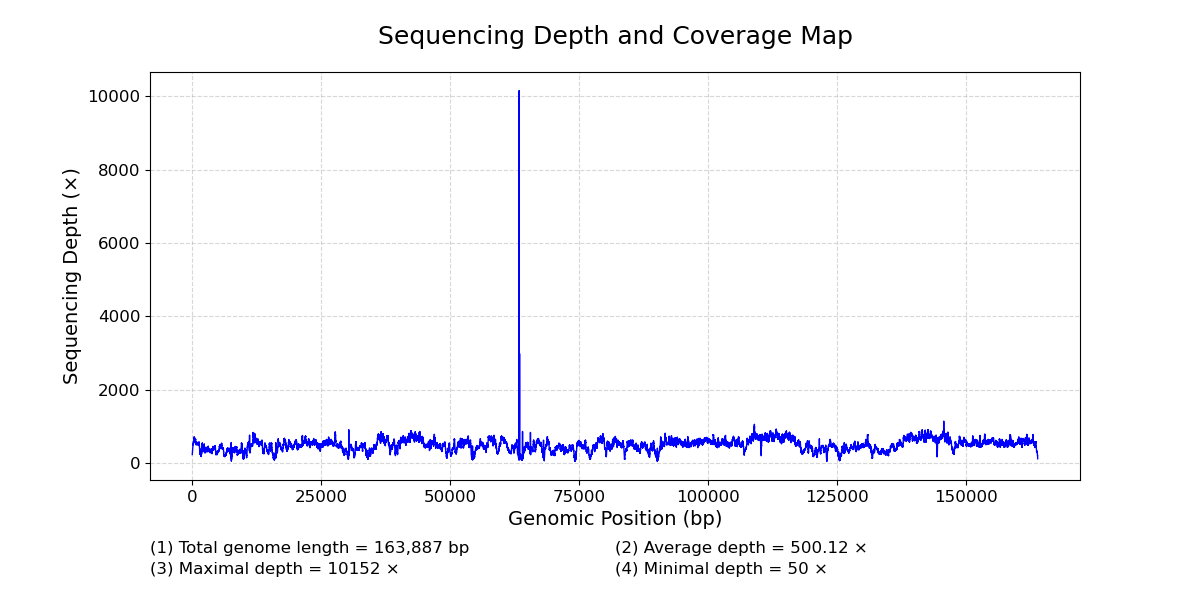


Supplementary Figure S1. Coverage map of chloroplast genome of *Callisia fragrans*.


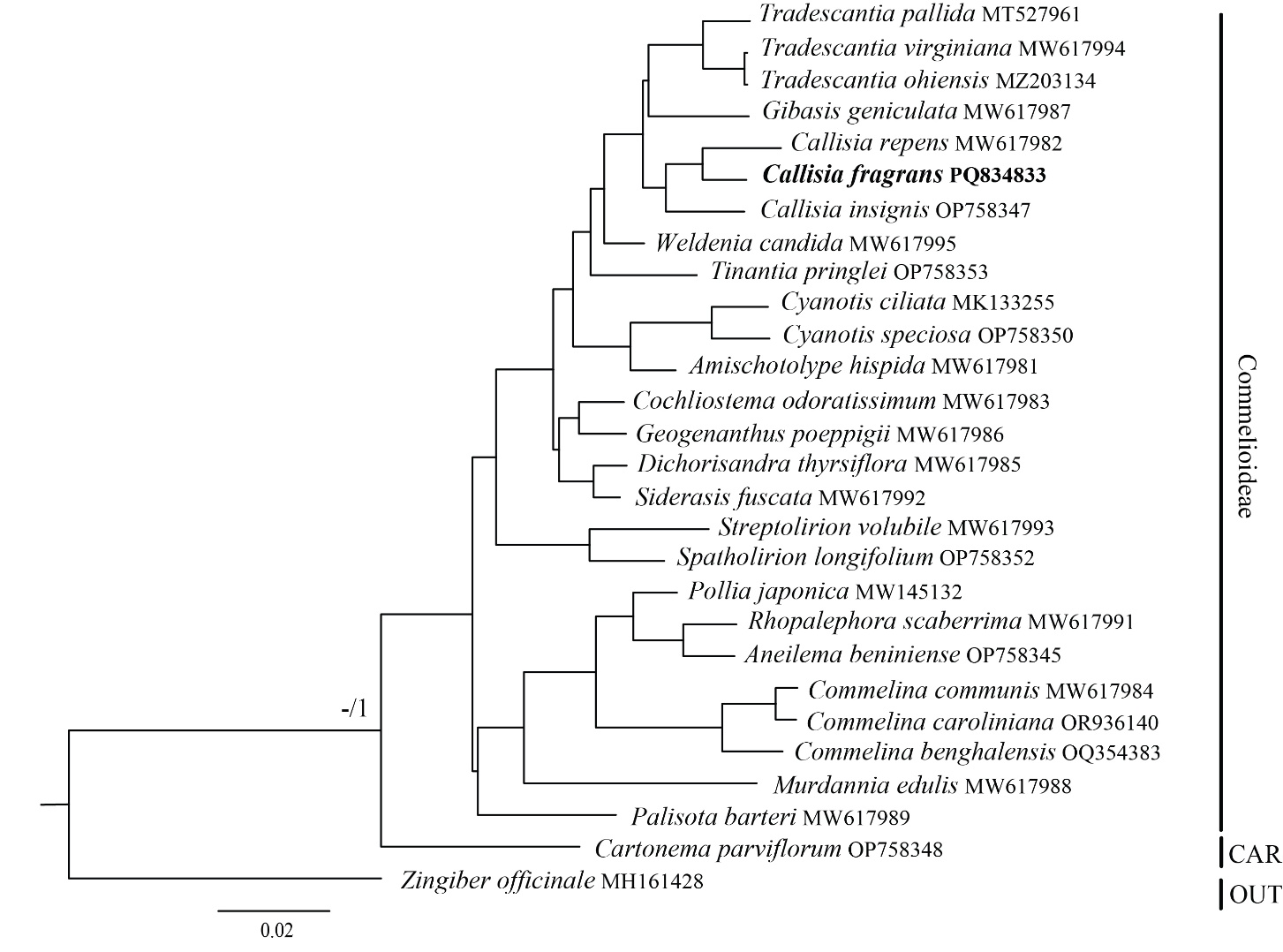


**Supplementary Figure S2.** Phylogenetic relationship of *Callisia fragrans* and related species inferred from whole chloroplast genome sequences using maximum likelihood and Bayesian Inference methods. Only bootstrap values smaller than 100 and posterior probability under 1 were shown at the nodes. The bold italic name means the newly generated chloroplast genome in this study. CAR: Cartonematoideae; OUT: Outgroup.
